# Comparison of nutrient profiling models for assessing the nutritional quality of foods: a validation study

**DOI:** 10.1017/S0007114518001575

**Published:** 2018-07-17

**Authors:** Theresa Poon, Marie-Ève Labonté, Christine Mulligan, Mavra Ahmed, Kacie M. Dickinson, Mary R. L’Abbé

**Affiliations:** 1 Department of Nutritional Sciences, Faculty of Medicine, University of Toronto, FitzGerald Building, 150 College Street, Toronto, ON, Canada M5S 3E2; 2 Institute of Nutrition and Functional Foods, Laval University, 2440 Hochelaga Boulevard, Québec City, QC, Canada G1V 0A6; 3 Nutrition and Dietetics, College of Nursing and Health Sciences, Flinders University, GPO Box 2100, Adelaide, SA 5001, Australia

**Keywords:** Nutrient profiling, Nutritional quality, Healthfulness, Validation, Content validity, Construct validity, Convergent validity

## Abstract

Nutrient profiling (NP) is a method for evaluating the healthfulness of foods. Although many NP models exist, most have not been validated. This study aimed to examine the content and construct/convergent validity of five models from different regions: Australia/New Zealand (FSANZ), France (Nutri-Score), Canada (HCST), Europe (EURO) and Americas (PAHO). Using data from the 2013 UofT Food Label Information Program (*n*15342 foods/beverages), construct/convergent validity was assessed by comparing the classifications of foods determined by each model to a previously validated model, which served as the reference (Ofcom). The parameters assessed included associations (Cochran–Armitage trend test), agreement (*κ* statistic) and discordant classifications (McNemar’s test). Analyses were conducted across all foods and by food category. On the basis of the nutrients/components considered by each model, all models exhibited moderate content validity. Although positive associations were observed between each model and Ofcom (all *P*_trend_<0·001), agreement with Ofcom was ‘near perfect’ for FSANZ (*κ*=0·89) and Nutri-Score (*κ*=0·83), ‘moderate’ for EURO (*κ*=0·54) and ‘fair’ for PAHO (*κ*=0·28) and HCST (*κ*=0·26). There were discordant classifications with Ofcom for 5·3 % (FSANZ), 8·3 % (Nutri-Score), 22·0 % (EURO), 33·4 % (PAHO) and 37·0 % (HCST) of foods (all *P*<0·001). Construct/convergent validity was confirmed between FSANZ and Nutri-Score *v.* Ofcom, and to a lesser extent between EURO *v.* Ofcom. Numerous incongruencies with Ofcom were identified for HCST and PAHO, which highlights the importance of examining classifications across food categories, the level at which differences between models become apparent. These results may be informative for regulators seeking to adapt and validate existing models for use in country-specific applications.

Nutrient profiling (NP), defined as the science of classifying foods according to their nutritional composition for the purpose of promoting health and preventing disease, is a relatively new term in the field of nutrition research^(^ ^1^ ^,^ ^2^ ^)^. Several of the earliest forms of NP were introduced by government bodies in the 1980s and 1990s, including the US Special Supplemental Nutrition Program for Women, Infants, and Children in 1980^(^ ^3^ ^)^, Swedish Keyhole in 1989^(^ ^4^ ^)^ and disqualifying nutrient levels for US health claims in 1993^(^ ^5^ ^)^. The term NP gained ground following the development of the Ofcom model by the UK Food Standards Agency in 2004 to 2005^(^ ^6^ ^,^ ^7^ ^)^ and the mention of nutrient profiles in Regulation (EC) No 1924/2006 on nutrition and health claims by the European Commission in 2006^(^ ^8^ ^)^. In 2010, NP became even more widely known when the WHO provided its Member States with a set of recommendations on the marketing of foods and beverages to children, one of which advocated the use of NP models for defining the products to be covered by the marketing restrictions^(^ ^9^ ^)^. Globally, NP is now recognised as a transparent and reproducible method of evaluating the healthfulness of foods, and for its use in numerous applications in government and industry (e.g. front-of-pack food labelling, food taxes, reformulation)^(^ ^10^ ^,^ ^11^ ^)^.

The number of potential NP models identified globally was thirty-nine (included models only) on the basis of a systematic review conducted in 2008^(^ ^12^ ^)^, and 387 on the basis of a systematic review conducted in 2016^(^ ^13^ ^)^. Given this recent proliferation of NP models and the extensive resources required to develop and validate a new model, the adaptation of an existing model is preferred by the WHO and is becoming increasingly common practice for government agencies^(^ ^1^ ^,^ ^11^ ^)^. It is prudent to adapt a model that has been developed by an authoritative body and, more importantly, validated^(^ ^1^ ^,^ ^11^ ^,^ ^14^ ^)^. However, although many models exist, the majority of NP models have not been fully evaluated or validated before their implementation^(^ ^11^ ^,^ ^14^ ^)^. This is partly owing to the infancy of the methods used to validate NP models^(^ ^11^ ^)^. Therefore, several researchers have suggested that validity testing of NP models should be given the highest priority in this field of research^(^ ^10^ ^,^ ^14^ ^,^ ^15^ ^)^.

There are several types of validity (e.g. criterion, convergent, construct, content), and there are different methods of testing for validity^(^ ^1^ ^,^ ^16^ ^)^. Content validity is defined as the extent to which the model encompasses the full range of meaning for the concept being measured^(^ ^16^ ^)^. One method of testing for content validity is to assess the consistency between the algorithmic underpinnings of a model and the current scientific literature^(^ ^16^ ^)^, such as whether a model considers nutrients of public health concern (e.g. Na).

Different variations of the definitions for criterion, convergent and construct validity have been used by different researchers of NP validation (online Supplementary Table S1). The definitions of criterion validity used by various researchers differ with regard to the necessity of the use of a ‘gold-standard’ comparator in NP^(^ ^14^ ^,^ ^16^ ^–^ ^18^ ^)^. In addition, the term concurrent validity (i.e. a type of criterion validity that requires the measurement and criterion to refer to the same point in time^(^ ^14^ ^)^) is rarely used in the literature on NP, whereas the term convergent validity is more widely used^(^ ^16^ ^,^ ^17^ ^)^. On the basis of the similarities between the definitions for criterion and concurrent *v.* convergent validity, it appears that convergent validity should be used particularly when a comparison with a non-‘gold standard’ is conducted. Although there is commonality across these definitions used by different researchers, this inconsistency in the terminology used has led to ambiguity with regard to the type of validity that can be inferred from using certain methods. For example, criterion^(^ ^16^ ^)^, convergent^(^ ^16^ ^)^ and construct^(^ ^17^ ^)^ validity have all been used to describe the extent to which the method correlates in a predicted manner with theoretical concepts. Similarly, both convergent^(^ ^17^ ^)^ and construct^(^ ^14^ ^,^ ^19^ ^)^ validity have been used to describe the comparison with other measures of the same or a closely related variable. Thus, on the basis of these observations, the terms construct/convergent validity are used in this study because they have been used previously to describe the comparisons of NP models with a non-‘gold-standard’ model that has been validated.

The intent for this study was 2-fold. First, as it is prudent to adapt a model that has been developed by an authoritative body^(^ ^1^ ^,^ ^11^ ^)^, only models developed by authoritative bodies were considered in this study (e.g. models developed by industry were not considered). Second, only a single study related to the use of an NP model in a Canadian context was identified; however, in this study, only the face validity (i.e. extent to which the system is a useful tool for end users^(^ ^16^ ^)^) of the Health Canada Surveillance Tool (HCST) tier system in assessing the nutritional quality of Canadian diets was discussed^(^ ^20^ ^)^. To our knowledge, there have been no additional studies that examined any type of validity of any other model when applied to the Canadian food supply. As such, this study focused on models applicable to the Canadian Westernised food supply (e.g. models from regions that were not relevant to a North American context owing to differences in food supplies were not considered). Thus, the objective of this study was to examine the content and construct/convergent validity of a variety of NP models developed by authoritative bodies applicable to the Canadian context in assessing the nutritional quality of pre-packaged foods from a large, national, branded database.

## Methods

### Nutrient profiling models

Several NP models developed by authoritative bodies applicable to the Canadian Westernised food supply were selected for this study, including the HCST tier system^(^ ^21^ ^)^, which is currently the only NP model developed by the federal institution responsible for the health of Canadians (i.e. Health Canada). In addition, the models developed by the authoritative bodies in the UK^(^ ^22^ ^)^, Australia and New Zealand^(^ ^23^ ^)^ and France^(^ ^24^ ^)^ were retained as these countries were considered to have food supplies similar to that in Canada. Moreover, two international models developed by the regional offices of the WHO for Europe^(^ ^25^ ^)^ and the Americas^(^ ^26^ ^)^ were retained for their wide applicability, given that they were developed or tested for use in several countries considered to have food supplies similar to that in Canada. The key characteristics of the models examined in this study are summarised in Table 1 and described below.

**Table 1:** Summary of nutrient profiling (NP) models examined

	Ofcom (reference*)	FSANZ	Nutri-Score	HCST	EURO	PAHO
Food categories (*n*)	2	3	2	4	20†	5
Reference amount	100 g‡	100 g or ml	100 g	Serving	100 g	% energy of food§
Nutrients/food components (*n*)	7	7	7	4	8	6
Energy||	✓	✓	✓		✓	
Total fat||				✓	✓	✓
Saturated fat||	✓	✓	✓	✓	✓	✓
Unsaturated fat¶						
*Trans*-fat||					✓ (industrially produced)**	✓ (total)
Na||	✓	✓	✓	✓	✓	✓
Total sugars||	✓	✓	✓	✓	✓	
Free/added sugars||					✓ (added)††	✓ (free)
Sweeteners					✓	✓
Protein	**✓**	**✓**	**✓**			
Fibre	**✓**	**✓**	**✓**			
Fruits/vegetables¶	✓ (FVNL)‡‡	✓ (FVNL)‡‡	✓ (FVNL)‡‡			
Continuous outcome	✓ (Ofcom score)	✓ (FSANZ score)	✓ (Nutri-Score score)			
Ordinal outcome	✓ (Ofcom quartile)	✓ (FSANZ quartile)	✓ (Nutri-Score class A to E)	✓ (HCST tier 1 to 4)		
Dichotomous outcome	✓	✓			✓	✓
‘Healthier’ foods	Permitted for marketing to children	Permitted to carry health claims	Dark green (A) and light green (B)§§	Tier 1 and 2 foods in line with CFG§§	Permitted for marketing to children	Not excessive in any nutrient
‘Less healthy’ foods	Not permitted for marketing to children	Not permitted to carry health claims	Yellow (C), light orange (D) and dark orange (E)§§	Tier 3 foods partially in line with CFG and tier 4 foods not in line with CFG§§	Not permitted for marketing to children	Excessive in ≥1 nutrient(s)

FSANZ, Food Standards Australia New Zealand; HCST, Health Canada Surveillance Tool; EURO, WHO Regional Office for Europe; PAHO, WHO Regional Office for the Americas/Pan American Health Organization; FVNL, fruits, vegetables, nuts and legumes; CFG, Canada’s Food Guide.

* The Ofcom model was chosen as the reference model for the assessment of construct/convergent validity in this study.

† The model consists of seventeen main categories, with the beverage category containing four sub-categories, for a total of twenty unique categories: (1) chocolate and sugar confectionery, energy bars and sweet toppings and desserts; (2) cakes, sweet biscuits, pastries, other sweet bakery wares and dry mixes for making such; (3) savoury snacks; (4) beverages: juices; (5) beverages: milk drinks; (6) beverages: energy drinks; (7) beverages: other beverages; (8) edible ice; (9) breakfast cereals; (10) yogurts, sour milk, cream and other similar foods; (11) cheese; (12) ready-made and convenience foods and composite dishes; (13) butter and other fats and oils; (14) bread, bread products and crisp breads; (15) fresh or dried pasta, rice and grains; (16) fresh and frozen meat, poultry, fish and similar; (17) processed meat, poultry, fish and similar; (18) fresh and frozen fruit, vegetables and legumes; (19) processed fruit, vegetables and legumes; and (20) sauces, dips and dressings^(^ ^25^ ^)^.

‡ Although a reference amount of 100 g for foods and beverages was specified as part of the model, a reference amount of 100 ml for beverages and other products in liquid form was used for the assessment of construct/convergent validity in this study.

§ While sweeteners were evaluated based on their absence or presence in the ingredient list and Na was evaluated on a per kJ (kcal) basis, the thresholds for the other nutrients were presented as a % energy of the food (e.g. an excess of total fat is ≥30 % of total energy of the food)^(^ ^26^ ^)^.

|| For the assessment of content validity in this study, the nutrients to limit according to the WHO include energy, total fat, saturated fat, *trans*-fat, Na and sugars (type not specified)^(^ ^27^ ^,^ ^28^ ^)^.

¶ For the assessment of content validity in this study, the nutrients to encourage according to the WHO include unsaturated fat and fruits and vegetables^(^ ^27^ ^,^ ^28^ ^)^.

** For the assessment of construct/convergent validity in this study, the industrially produced *trans*-fat content of a food was estimated on the basis of the presence of hydrogenated or partially hydrogenated oils in the ingredient list and the total *trans*-fat level declared in the Nutrition Facts table.

†† Although added sugars were specified as part of the model, free sugar levels estimated using the University of Toronto’s free sugar algorithm^(^ ^29^ ^)^ were used for the assessment of construct/convergent validity in this study.

‡‡ For the assessment of construct/convergent validity in this study, the FVNL content of a food was estimated based on the presence and positions of the FVNL ingredients within the ingredient list.

§§ As the Nutri-Score and HCST models do not generate dichotomous outcomes, the authors provided the definitions for ‘healthier’ and ‘less healthy’ foods in this study.

The Ofcom model was developed for the regulation of television advertising to children in the UK^(^ ^22^ ^)^. The model consists of two food categories: (1) beverages and (2) foods. It takes into consideration a total of seven nutrients to limit and nutrients/food components to encourage, the latter including fruit, vegetable, nut and legume (FVNL) content. To estimate the FVNL content of a food without quantitative declarations in the ingredient list, which are not required in Canada, the presence and positions of the FVNL ingredients within the ingredient list were used (online Supplementary Table S2). On the basis of the level of nutrients/components present per 100 g of the food, the model generates a summary score in which a lower score represents a food with a more favourable nutritional profile. The model also classifies the food as ‘permitted’ or ‘not permitted’ for advertising to children based on pre-determined cut-off scores for foods and beverages.

The Food Standards Australia New Zealand (FSANZ) model was developed for the regulation of health claims on foods in Australia and New Zealand^(^ ^23^ ^)^. As a derivative of the Ofcom model, the FSANZ model is similar, except for the following characteristics: it consists of an additional food category of cheeses high in Ca (i.e. >320 mg/100 g) and fats; it considers nutrients on a per 100-ml basis in addition to 100 g; and its FVNL definition differs slightly (e.g. inclusion of potatoes and tubers). The method used to estimate the FVNL content for the FSANZ model was similar to that used to estimate the FVNL content for the Ofcom model, and is described in detail elsewhere^(^ ^30^ ^)^. The model classifies the food as ‘permitted’ or ‘not permitted’ to carry health claims based on pre-determined cut-off scores for each food category.

The Nutri-Score model was developed for use in front-of-pack food labelling and food reformulation in France^(^ ^24^ ^)^. As a derivative of the Ofcom model, the Nutri-Score model is similar, except that alternative nutrient criteria and/or pre-determined cut-off scores are considered for certain cheeses, fats and beverages. On the basis of the level of nutrients/components present per 100 g of the food and its food category, the model generates a summary score in which a lower score represents a food with a more favourable nutritional profile. In addition, the model classifies the food into one of five classes, each associated with a colour and letter, ranging from higher to lower nutritional quality as follows: dark green (A), light green (B), yellow (C), light orange (D) and dark orange (E).

The HCST tier system was developed to assess the adherence of the dietary intakes of Canadians to the dietary guidance provided by Canada’s Food Guide (CFG)^(^ ^21^ ^)^. The model consists of four food categories: (1) vegetables and fruits; (2) grain products; (3) milk and alternatives; and (4) meat and alternatives. It takes into consideration four nutrients to limit (i.e. total fat, saturated fat, Na and total sugars). On the basis of the level of nutrients present per serving of the food, the model classifies the food into one of four tiers as follows: tier 1 and 2 foods are in line with CFG and should be consumed often; tier 3 foods are partially in line with CFG and should be consumed less often; and tier 4 foods are not in line with CFG and their consumption should be limited.

The WHO Regional Office for Europe (EURO) model was developed for the restriction of marketing unhealthy foods to children^(^ ^25^ ^)^. The model consists of twenty food categories (listed in a footnote to Table 1). It takes into consideration eight nutrients to limit, including industrially produced *trans*-fat and added sugars. Given that quantitative declarations of industrially produced *trans*-fat are not required in Canada, the amount was estimated on the basis of the presence of hydrogenated or partially hydrogenated oils in the ingredient list and the total *trans*-fat level declared in the Nutrition Facts table. Instead of added sugars, free sugar levels were used in this study because the WHO considers free sugars as part of their guidelines on sugars^(^ ^31^ ^)^. To estimate the free sugar content of a food without a quantitative declaration in the Nutrition Facts table, which is not required in Canada, the University of Toronto’s free sugar algorithm was used; this algorithm is described elsewhere^(^ ^29^ ^)^. On the basis of the level of nutrients present per 100 g of the food, the model classifies the food as ‘permitted’ or ‘not permitted’ for marketing to children. It should be noted that seven food categories are not subject to the nutrient criteria and are automatically classified as ‘permitted’ (i.e. fresh and frozen meat/poultry/fish; fresh and frozen fruits/vegetables/legumes) or ‘not permitted’ for marketing (i.e. confectionery; sweet bakery products; juices; energy drinks; edible ice).

The WHO Regional Office for the Americas/Pan American Health Organization (PAHO) model was developed for a variety of regulatory purposes aimed at addressing the obesity epidemic^(^ ^26^ ^)^. The model consists of five food categories: (1) ultra-processed foods; (2) processed foods; (3) unprocessed or minimally processed foods; (4) culinary ingredients; and (5) freshly prepared dishes. It takes into consideration six nutrients to limit. On the basis of the level of nutrients present per % energy of the food, the model classifies the food as ‘not excessive’ in any nutrient or ‘excessive’ in one or more of the nutrients. It should be noted that three food categories (i.e. unprocessed or minimally processed foods; culinary ingredients; and freshly prepared dishes) are not subject to the nutrient criteria and are automatically classified as ‘not excessive’ in any nutrient.

In this study, ‘healthier’ foods were defined as those permitted for marketing to children as per the Ofcom and EURO models; permitted to carry health claims as per FSANZ; classified as dark green (A) or light green (B) as per Nutri-Score; classified as tier 1 or 2 as per HCST; and not excessive in any nutrient as per PAHO (Table 1). Correspondingly, ‘less healthy’ foods were defined as those not permitted for marketing to children as per the Ofcom and EURO models; not permitted to carry health claims as per FSANZ; classified as yellow (C), light orange (D) or dark orange (E) as per Nutri-Score; classified as tier 3 or 4 as per HCST; and excessive in one or more nutrients as per PAHO.

### Content validity

For the FSANZ, Nutri-Score, HCST, EURO and PAHO models, content validity was assessed by examining the consistency between the nutrients/food components included in the models *v.* those considered within the WHO’s *Global Action Plan for the Prevention and Control of Non-Communicable Disease 2013–2020*, which was used because it was recently updated in May 2017 and represents consensus in the current scientific literature with regard to the nutrients/components of immediate importance in promoting health and preventing disease^(^ ^27^ ^,^ ^28^ ^)^. These included the nutrients/components explicitly stated within objective three of the action plan as those to encourage (i.e. unsaturated fat and fruits and vegetables) and those to limit (i.e. energy, total fat, saturated fat, *trans*-fat, Na and sugars (type not specified))^(^ ^27^ ^,^ ^28^ ^)^.

### Construct/convergent validity

Construct/convergent validity was assessed by comparing the classifications of foods determined by each of the models (FSANZ, Nutri-Score, HCST, EURO and PAHO) *v.* those determined by a reference model using several parameters. The Ofcom model was chosen as the reference model because it has been extensively validated using various methods for different applications in multiple countries^(^ ^10^ ^)^. Specifically, the Ofcom model has been shown to have content^(^ ^6^ ^,^ ^7^ ^,^ ^32^ ^)^, construct^(^ ^14^ ^,^ ^17^ ^)^, convergent^(^ ^17^ ^)^, discriminant^(^ ^17^ ^)^ and concurrent validity^(^ ^18^ ^)^. In addition, the adaptation of the Ofcom model into a dietary index to represent the overall quality of an individual’s diet has been demonstrated to have predictive validity^(^ ^33^ ^–^ ^37^ ^)^. Details of the food database and parameters used in this assessment are provided below.

### Food database

A total of 15 342 pre-packaged foods and beverages from the University of Toronto’s Food Label Information Program (FLIP) 2013 database were examined. Data were collected in 2013 across the four largest grocery chains in Canada, which represented 75·4 % of the grocery retail market share^(^ ^29^ ^)^. The product information collected included the Nutrition Facts table and ingredient list, among other data. Details of FLIP 2013 are provided elsewhere^(^ ^29^ ^)^. Foods were classified into the twenty-two food categories as per Schedule M of the *Food and Drug Regulations* (version in force between March 2012 and December 2016)^(^ ^38^ ^)^. A total of 115 products were excluded from the analyses: fifty-five products owing to manufacturer errors in the nutrient declarations in the Nutrition Facts table (i.e. >20 % difference between the energy content declared and energy content calculated using Atwater factors for macronutrients) and sixty products that did not align with any Schedule M category (i.e. fifty-five meal replacements, four instant or dry yeast products and one natural health product). Thus, 15 227 unique products were available for analyses. To generate the classifications of the foods for each NP model, the 15 227 foods in FLIP 2013 were first classified independently by two authors (M. A. and K. M. D. for Nutri-Score; M.-E. L. and C. M. for HCST; and T. P. and M.-E. L. for other models) into the food categories specific to the NP models using information from the ingredient lists and/or pre-classifications from the Schedule M food categories or sugar-focused categories from Bernstein *et al.* ^(^ ^29^ ^)^. For all models, nutrient data for products in their ‘as consumed’ form were used. Subsequently, the nutrient criteria for each model were applied to the foods.

### Statistical analyses

Construct/convergent validity was assessed using five parameters. First, the association between the proportion and 95 % CI of foods classified as ‘less healthy’ by each model and quartiles of Ofcom scores was assessed across all foods using the Cochran–Armitage trend test. Second, pairwise agreement between each model and the Ofcom model in the proportions of foods classified as ‘healthier’ or ‘less healthy’ was assessed across all foods (*n* 15 227) and by the twenty-two Schedule M food categories using the *κ* statistic (95 % CI), as follows: 0·01–0·20 ‘slight’; 0·21–0·40 ‘fair’; 0·41–0·60 ‘moderate’; 0·61–0·80 ‘substantial’; and 0·81–0·99 ‘near perfect’^(^ ^39^ ^)^. Third, discordant classifications (hereafter referred to as ‘discordance’) between each model and Ofcom were defined as the sum of the percentage of foods classified as ‘healthier’ by a model but ‘less healthy’ by Ofcom and the percentage of foods classified as ‘less healthy’ by a model but ‘healthier’ by Ofcom. Discordance was assessed across all foods and by food category using McNemar’s test for paired data.

Fourth, because the FSANZ and Nutri-Score models also generated continuous outcomes (Table 1), Pearson’s correlation coefficients were determined between FSANZ scores and Nutri-Score scores *v.* Ofcom scores. Fifth, because the FSANZ, Nutri-Score and HCST models also generated ordinal outcomes (Table 1), cross-classification analyses were conducted between (1) quartiles of FSANZ scores and the four HCST tiers *v.* quartiles of Ofcom scores, and (2) the five Nutri-Score classes *v.* quintiles of Ofcom scores. Exact agreement was defined as the classification of a food in the same quartiles/tiers or classes/quintiles using different models (e.g. FSANZ quartile 1 and Ofcom quartile 1; Nutri-Score B and Ofcom quintile 2; HCST tier 4 and Ofcom quartile 4). In addition, agreement within an adjacent (i.e. ±1) quartile/tier or class/quintile (e.g. FSANZ quartile 1 and Ofcom quartile 2; Nutri-Score B and Ofcom quintile 1; HCST tier 4 and Ofcom quartile 3) and disagreement (e.g. FSANZ quartile 1 and Ofcom quartile 3; Nutri-Score B and Ofcom quintile 4; HCST tier 4 and Ofcom quartile 2) were assessed. Last, gross misclassification was defined as the classification of a food in opposite quartiles/tiers or classes/quintiles (e.g. FSANZ quartile 1 and Ofcom quartile 4; Nutri-Score A and Ofcom quintile 5; HCST tier 4 and Ofcom quartile 1). A *P* value <0·05 was considered statistically significant. Statistical analyses were conducted using SAS 9.4 (SAS Institute Inc.).

## Results

### Content validity

Of the eight nutrients/food components considered by the WHO to be of immediate importance in promoting health and preventing disease (i.e. energy, total fat, saturated fat, unsaturated fat, *trans*-fat, Na, sugars (type not specified) and fruits and vegetables)^(^ ^27^ ^,^ ^28^ ^)^, each of the NP models considered at least four of these nutrients/components (Table 1). With respect to the nutrients to limit, saturated fat, Na and some form of sugar (e.g. total, free or added) were all considered in each of the five models. Total fat was considered in the HCST, EURO and PAHO models; energy was considered in FSANZ, Nutri-Score and EURO; and *trans*-fat was considered in EURO and PAHO. With respect to the nutrients/components to encourage, the fruit and vegetable content of foods was considered in the FSANZ and Nutri-Score models, and unsaturated fat was not considered in any model.

### Construct/convergent validity

**Fig. 1:**
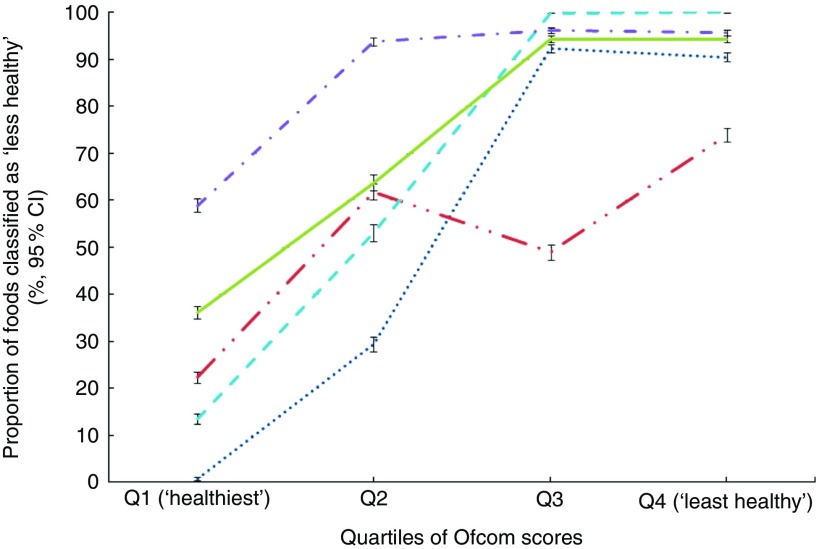
Overall associations between the proportions (%, 95 % CI) of foods classified as ‘less healthy’ by the models and quartiles of Ofcom scores (*n* 15 227; all *P*<0·001 for trend using the Cochran–Armitage trend test). Data were missing for 0·29–0·41 % (*n* 44 to 62) of foods across the comparisons with the Ofcom model. 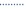, Food Standards Australia New Zealand; 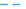, Nutri-Score; 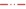, Health Canada Surveillance Tool; 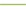, WHO Regional Office for Europe; 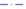, WHO Regional Office for the Americas/Pan American Health Organization.

Across all foods, a positive association was observed between each model and the Ofcom model, such that the proportion of foods classified as ‘less healthy’ by FSANZ, Nutri-Score, HCST, EURO or PAHO increased across quartiles of Ofcom scores, with the highest quartile representing the ‘least healthy’ foods (all *P*<0·001 for trend) (Fig. 1). However, varying levels of agreement and discordance were observed between each model and Ofcom, as described below.

### Food Standards Australia New Zealand model

**Fig. 2:**
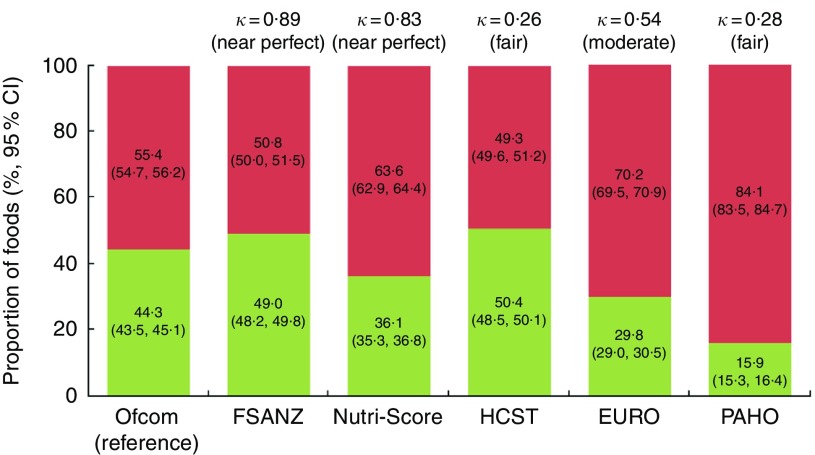
Overall proportions (%, 95 % CI) of ‘healthier (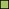)’ and ‘less healthy (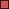)’ foods and agreement (*κ*) between each model and the Ofcom model (*n* 15 227). Data were missing for 0·01–0·34 % (*n* 1 to 52) of foods within a model. Agreement was assessed using the *κ* statistic as follows: 0·01–0·20 ‘slight’; 0·21–0·40 ‘fair’; 0·41–0·60 ‘moderate’; 0·61–0·80 ‘substantial’; 0·81–0·99 ‘near perfect’^(^ ^39^ ^)^. FSANZ, Food Standards Australia New Zealand; HCST, Health Canada Surveillance Tool; EURO, WHO Regional Office for Europe; PAHO, WHO Regional Office for the Americas/Pan American Health Organization.

**Fig. 3:**
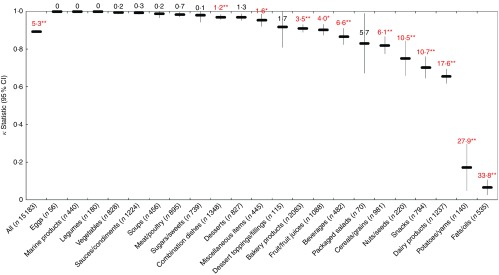
Agreement (*κ*, 95 % CI) and discordance (%, indicated above each line) between the Food Standards Australia New Zealand (FSANZ) and Ofcom model for all foods (*n* 15 183; data missing for *n* 44) and twenty-two food categories from Schedule M of the *Food and Drug Regulations* ^(^ ^38^ ^)^. Agreement was assessed using the *κ* statistic as follows: 0·01–0·20 ‘slight’; 0·21–0·40 ‘fair’; 0·41–0·60 ‘moderate’; 0·61–0·80 ‘substantial’; 0·81–0·99 ‘near perfect’^(^ ^39^ ^)^. Significant discordance in classifications between models using McNemar’s test (* *P*<0·05, ** *P*<0·001).

Across all foods, there was ‘near perfect’ agreement (*κ*=0·89; 95 % CI 0·89, 0·90) between the FSANZ and Ofcom model. Although the overall proportions of foods classified as ‘healthier’ by the two models were similar in magnitude (49·0 % by FSANZ; 44·3 % by Ofcom), significant discordance in the classifications between the models was observed for 5·3 % of the large sample of 15 183 foods analysed (*P*<0·001) (Figs 2 and 3). Specifically, significant discordance was observed for eleven of the twenty-two food categories analysed (all *P*<0·05); however, agreement was ‘near perfect’ or ‘substantial’ for nine of these categories (*κ*=0·66 to 0·97; 1·2 to 17·6 % discordance). Notably, among the remaining two categories, ‘slight’ agreement and higher proportions of discordance were observed: potatoes/sweet potatoes/yams (*κ*=0·17; 27·9 % discordance) and fats/oils (*κ*=0·07; 33·8 % discordance).

Across all foods, the FSANZ and Ofcom scores were positively correlated (Pearson’s *r*=0·973; *P*<0·001). Cross-classification analyses conducted between quartiles of FSANZ and Ofcom scores indicated that the classifications were in exact agreement for 95·2 %, within an adjacent quartile for 4·2 %, and in disagreement for 0·3 % of foods, and that there was no gross misclassification of foods (data were missing for 0·3 % of foods; data across food categories not shown).

### Nutri-Score model

**Fig. 4:**
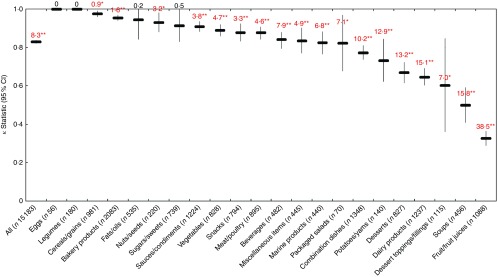
Agreement (*κ*, 95 % CI) and discordance (%, indicated above each line) between the Nutri-Score and Ofcom model for all foods (*n* 15 183; data missing for *n* 44) and twenty-two food categories from Schedule M of the *Food and Drug Regulations* ^(^ ^38^ ^)^. Agreement was assessed using the *κ* statistic as follows: 0·01–0·20 ‘slight’; 0·21–0·40 ‘fair’; 0·41–0·60 ‘moderate’; 0·61–0·80 ‘substantial’; 0·81–0·99 ‘near perfect’^(^ ^39^ ^)^. Significant discordance in classifications between models using McNemar’s test (* *P*<0·05, ** *P*<0·001).

Across all foods, there was ‘near perfect’ agreement (*κ*=0·83; 95 % CI 0·82, 0·84) between the Nutri-Score and Ofcom model. The overall proportions of foods classified as ‘healthier’ by the two models differed (36·1 % by Nutri-Score; 44·3 % by Ofcom), and significant discordance in the classifications between the models was observed for 8·3 % of the foods (*P*<0·001) (Figs. 2 and 4). With the exception of eggs and legumes (for which there was no discordance), as well as fats/oils (*P*=0·32) and sugars/sweets (*P*=0·05), significant discordance was observed for eighteen of the twenty-two food categories analysed (all *P*<0·05). However, agreement was ‘near perfect’, ‘substantial’ or ‘moderate’ for seventeen of these categories (*κ*=0·50 to 0·98; 0·9 to 15·8 % discordance). Notably, ‘fair’ agreement and a higher proportion of discordance was observed in the remaining category for fruit/fruit juices (*κ*=0·33; 38·5 % discordance).

**Fig. 5:**
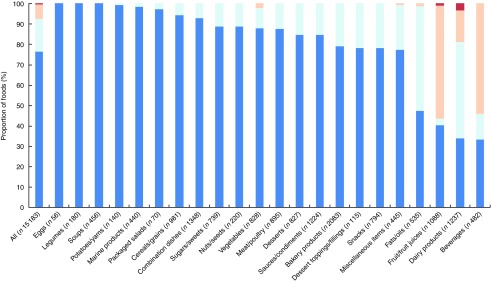
Cross-classification analyses between five Nutri-Score classes *v.* quintiles of Ofcom scores for all foods (*n* 15 183; data missing for *n* 44) and twenty-two food categories from Schedule M of the *Food and Drug Regulations* ^(^ ^38^ ^)^. Exact agreement occurs when a food is classified in the same classes/quintiles (e.g. Nutri-Score A and Ofcom quintile 1). Agreement within an adjacent (±1) class/quintile (e.g. Nutri-Score A and Ofcom quintile 2) and disagreement (e.g. Nutri-Score A and Ofcom quintile 3) also were assessed. Gross misclassification occurs when a food is classified in opposing classes/quintiles (e.g. Nutri-Score A and Ofcom quintile 5). 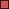, Gross misclassification; 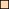, disagreement; 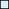, agreement ±1 class/quintile; 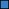, exact agreement.

Across all foods, the Nutri-Score and Ofcom scores were positively correlated (Pearson’s *r*=0·926; *P*<0·001). Cross-classification analyses conducted between the five Nutri-Score classes and quintiles of Ofcom scores indicated that the classifications were in exact agreement for 76·1 %, within an adjacent class/quintile for 16·1 %, in disagreement for 7·1 % or grossly misclassified for 0·3 % of foods (Fig. 5; data were missing for 0·3 % of foods). Specifically, the three food categories with the highest proportions of classifications that disagreed and/or were grossly misclassified included fruit/fruit juices (56·4 %), beverages (53·9 %) and dairy products (19·0 %). None of the categories had >5·0 % gross misclassification of foods.

### Health Canada Surveillance Tool tier system

**Fig. 6:**
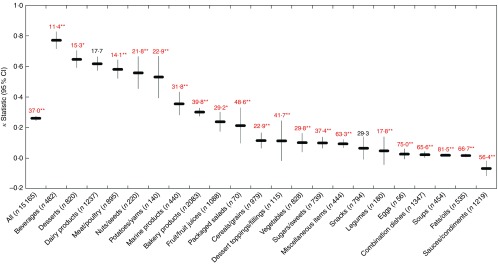
Agreement (*κ*, 95 % CI) and discordance (%, indicated above each line) between the Health Canada Surveillance Tool (HCST) and Ofcom model for all foods (*n* 15 165; data missing for *n* 62) and twenty-two food categories from Schedule M of the *Food and Drug Regulations* ^(^ ^38^ ^)^. Agreement was assessed using the *κ* statistic as follows: 0·01–0·20 ‘slight’; 0·21–0·40 ‘fair’; 0·41–0·60 ‘moderate’; 0·61–0·80 ‘substantial’; 0·81–0·99 ‘near perfect’^(^ ^39^ ^)^. Significant discordance in classifications between models using McNemar’s test (* *P*<0·05, ** *P*<0·001).

Across all foods, there was ‘fair’ agreement (*κ*=0·26; 95 % CI 0·25, 0·28) between the HCST and Ofcom model. Although the overall proportions of foods classified as ‘healthier’ by the two models were similar in magnitude (50·4 % by HCST; 44·3 % by Ofcom), significant discordance in the classifications between the models was observed for 37·0 % of the foods (*P*<0·001) (Figs 2 and 6). With the exception of dairy products/substitutes (*P*=0·38) and snacks (*P*=0·08), significant discordance was observed for twenty of the twenty-two food categories analysed (all *P*<0·05). However, agreement was ‘substantial’ or ‘moderate’ for five of these categories (*κ*=0·53 to 0·77; 11·4 to 22·9 % discordance). Among the remaining fifteen categories, less agreement and higher proportions of discordance were observed. Agreement was ‘fair’ or ‘slight’ (*κ*=0·02 for fats/oils to *κ*=0·36 for marine products), and discordance ranged from 17·8 % for legumes to 81·5 % for soups. Notably, there was systematic disagreement, as indicated by a negative *κ* statistic (*κ*=−0·07), and 56·4 % discordance for sauces/dips/gravies/condiments.

**Fig. 7:**
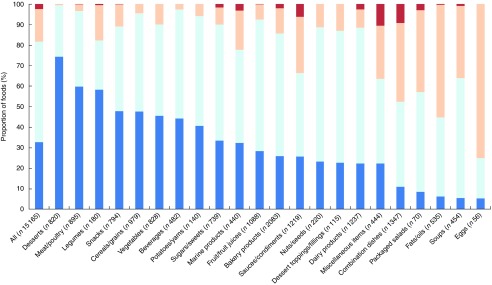
Cross-classification analyses between four Health Canada Surveillance Tool (HCST) tiers *v.* quartiles of Ofcom scores for all foods (*n* 15 165; data missing for *n* 62) and twenty-two food categories from Schedule M of the *Food and Drug Regulations* ^(^ ^38^ ^)^. Exact agreement occurs when a food is classified in the same tiers/quartiles (e.g. HCST tier 1 and Ofcom quartile 1). Agreement within an adjacent (±1) tier/quartile (e.g. HCST tier 1 and Ofcom quartile 2) and disagreement (e.g. HCST tier 1 and Ofcom quartile 3) also were assessed. Gross misclassification occurs when a food is classified in opposing tiers/quartiles (e.g. HCST tier 1 and Ofcom quartile 4). 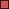, Gross misclassification; 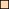, disagreement; 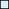, agreement ±1 tier/quartile; 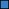, exact agreement.

Across all foods, cross-classification analyses conducted between the four HCST tiers and quartiles of Ofcom scores indicated that the classifications were in exact agreement for 32·6 %, within an adjacent tier/quartile for 48·8 %, in disagreement for 15·9 % or grossly misclassified for 2·3 % of foods (Fig. 7; data were missing for 0·4 % of foods). Specifically, the three food categories with the highest proportions of classifications that disagreed and/or were grossly misclassified included eggs (75·0 %), fats/oils (55·1 %) and combination dishes (47·5 %). A total of fifteen categories included grossly misclassified foods, with the highest proportions among miscellaneous items (10·6 %), combination dishes (9·2 %) and sauces/dips/gravies/condiments (6·2 %) (the remaining categories had <5·0 %).

### WHO Regional Office for Europe model

**Fig. 8:**
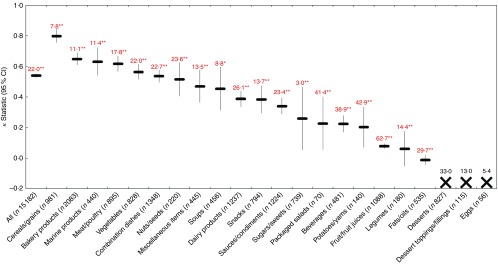
Agreement (*κ*, 95 % CI) and discordance (%, indicated above each line) between the WHO Regional Office for Europe (EURO) and Ofcom model for all foods (*n* 15 182; data missing for *n*45) and twenty-two food categories from Schedule M of the *Food and Drug Regulations* ^(^ ^38^ ^)^. Agreement was assessed using the *κ* statistic as follows: 0·01–0·20 ‘slight’; 0·21–0·40 ‘fair’; 0·41–0·60 ‘moderate’; 0·61–0·80 ‘substantial’; 0·81–0·99 ‘near perfect’^(^ ^39^ ^)^. Significant discordance in classifications between models using McNemar’s test (* *P*<0·01, ** *P*<0·001). The ‘X’ symbol represents a food category for which the *κ* statistic and McNemar’s test could not be conducted because 2×2 tables could not be generated (i.e. none of the desserts or dessert toppings/fillings was classified as ‘healthier’, and none of the eggs was classified as ‘less healthy’ by the EURO model).

Across all foods, there was ‘moderate’ agreement (*κ*=0·54; 95 % CI 0·53, 0·55) between the EURO and Ofcom model. The overall proportions of foods classified as ‘healthier’ by the two models differed (29·8 % by EURO; 44·3 % by Ofcom), and significant discordance in the classifications between the models was observed for 22·0 % of the foods (*P*<0·001) (Figs 2 and 8). According to the EURO model, none of the desserts or dessert toppings/fillings was classified as ‘healthier’, and none of the eggs was classified as ‘less healthy’ (online Supplementary Table S3); thus, the *κ* statistic and McNemar’s test for significance in discordance, which required 2×2 tables to be generated, could not be conducted for these three food categories. Significant discordance was observed for all nineteen food categories analysed using McNemar’s test (all *P*<0·01); however, agreement was ‘substantial’ or ‘moderate’ for nine of these categories (*κ*=0·45 to 0·80; 7·8 to 23·6 % discordance). Among the remaining ten categories, less agreement and higher proportions of discordance were observed. Agreement was ‘fair’ or ‘slight’ (*κ*=0·06 for legumes to *κ*=0·39 for dairy products/substitutes), and discordance ranged from 3·0 % for sugars/sweets to 62·7 % for fruit/fruit juices. Notably, there was systematic disagreement (*κ*=−0·01) and 29·7 % discordance for fats/oils.

### WHO Regional Office for the Americas/Pan American Health Organization model

**Fig. 9:**
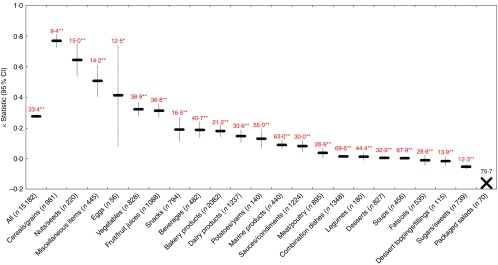
Agreement (*κ*, 95 % CI) and discordance (%, indicated above each line) between the WHO Regional Office for the Americas/Pan American Health Organization (PAHO) and Ofcom model for all foods (*n* 15 182; data missing for *n* 45) and twenty-two food categories from Schedule M of the *Food and Drug Regulations* ^(^ ^38^ ^)^. Agreement was assessed using the *κ* statistic as follows: 0·01–0·20 ‘slight’; 0·21–0·40 ‘fair’’; 0·41–0·60 ‘moderate’; 0·61–0·80 ‘substantial’; 0·81–0·99 ‘near perfect’^(^ ^39^ ^)^. Significant discordance in classifications between models using McNemar’s test (* *P*<0·01, ** *P*<0·001). The ‘X’ symbol represents a food category for which the *κ* statistic and McNemar’s test could not be conducted because 2×2 tables could not be generated (i.e. none of the packaged salads was classified as ‘healthier’ by the PAHO model).

Across all foods, there was ‘fair’ agreement (*κ*=0·28; 95 % CI 0·26, 0·29) between the PAHO and Ofcom model. The overall proportions of foods classified as ‘healthier’ by the two models differed (15·9 % by PAHO; 44·3 % by Ofcom), and significant discordance in the classifications between the models was observed for 33·4 % of the foods (*P*<0·001) (Figs 2 and 9). According to the PAHO model, none of the packaged salads (e.g. pasta or potato salads) was classified as ‘healthier’ (online Supplementary Table S3); thus, the *κ* statistic and McNemar’s test for significance in discordance, which required 2×2 tables to be generated, could not be conducted for this food category. Significant discordance was observed for all twenty-one food categories analysed using McNemar’s test (all *P*<0·01); however, agreement was ‘substantial’ or ‘moderate’ for four of these categories (*κ*=0·41–0·77; 9·4–15·0 % discordance). Among the remaining seventeen categories, less agreement and higher proportions of discordance were observed. Agreement was ‘fair’ or ‘slight’ (*κ*=0·01 for legumes to *κ*=0·32 for vegetables), and discordance ranged from 16·5 % for snacks to 69·6 % for combination dishes. Notably, there was no agreement (*κ*=0) or there was systematic disagreement (*κ*=−0·05 to −0·01), and a range of 12·3 to 87·9 % discordance for desserts, soups, fats/oils, dessert toppings/fillings and sugars/sweets.

The results of the assessment on the construct/convergent validity of the models are summarised in Table 2.

**Table 2:** Summary of results across the five parameters used to assess construct/convergent validity (*n* 15 227*)

		NP model				
Parameters	Statistical test or analysis	FSANZ	Nutri-Score	HCST	EURO	PAHO
Association between the proportion of ‘less healthy’ foods classified by model and quartiles of Ofcom scores	Cochran–Armitage trend test	Positive association (*P*<0·001)	Positive association (*P*<0·001)	Positive association (*P*<0·001)	Positive association (*P*<0·001)	Positive association (*P*<0·001)
Agreement with Ofcom	*κ* statistic†	Near perfect (*κ*=0·89)	Near perfect (*κ*=0·83)	Fair (*κ*=0·26)	Moderate (*κ*=0·54)	Fair (*κ*=0·28)
Discordance with Ofcom (% of foods)	McNemar’s test	5·3 (*P*<0·001)	8·3 (*P*<0·001)	37·0 (*P*<0·001)	22·0 (*P*<0·001)	33·4 (*P*<0·001)
Linear association between FSANZ and Nutri-Score scores *v.* Ofcom scores	Pearson’s correlation coefficient	*r*=0·973 (*P*<0·001)	*r*=0·926 (*P*<0·001)	N/A	N/A	N/A
Cross-classification analyses: agreement and misclassification between quartiles of FSANZ scores and 4 HCST tiers *v.* quartiles of Ofcom scores, or between 5 Nutri-Score classes *v.* quintiles of Ofcom scores (% of foods*)	Exact agreement	95·2	76·1	32·6	N/A	N/A
	Agreement ±1 quartile/tier or class/quintile	4·2	16·1	48·8		
	Disagreement	0·3	7·1	15·9		
	Gross misclassification	0	0·3	2·3		
Overall convergence with Ofcom		Convergent	Convergent	Not convergent	Moderately convergent	Not convergent

NP, nutrient profiling; FSANZ, Food Standards Australia New Zealand; HCST, Health Canada Surveillance Tool; EURO, WHO Regional Office for Europe; PAHO, WHO Regional Office for the Americas/Pan American Health Organization; N/A, not applicable.

* Across the different comparisons, data were missing for the following proportion of foods: FSANZ and Nutri-Score *v.* Ofcom, 0·29 % (*n* 44); HCST *v.* Ofcom, 0·41 % (*n* 62); EURO and PAHO *v.* Ofcom, 0·30 % (*n* 45).

† Agreement was assessed using the *κ* statistic as follows: 0·01–0·20 ‘slight’; 0·21–0·40 ‘fair’; 0·41–0·60 ‘moderate’; 0·61–0·80 ‘substantial’; 0·81–0·99 ‘near perfect’^(^ ^39^ ^)^.

## Discussion

In this study, the five NP models were considered to have moderate content validity in accounting for the nutrients/food components that characterise ‘healthy’ or ‘unhealthy’ diets according to the WHO^(^ ^27^ ^,^ ^28^ ^)^. Other studies that used this method of testing for content validity (i.e. assessing consistency between the underpinnings of a model and the current scientific literature^(^ ^16^ ^)^) were not identified in the published literature, possibly because content validity is typically assessed during the early phases of NP model development^(^ ^16^ ^)^ for which the data are rarely published. Several considerations should be noted. First, the WHO used the umbrella terms total fat and total sugars as part of the recommendations for avoiding an ‘unhealthy’ diet. Although recommendations specific to saturated, unsaturated and *trans*-fat also were provided, sugars were not further differentiated, despite the WHO’s consideration of free sugars as part of their sugar guidelines^(^ ^31^ ^)^. Second, a model’s inclusion of nutrients/components for which their roles in health are still being debated (e.g. sweeteners, saturated fat) was not considered to detract from its content validity. Third, a model’s lack of inclusion of nutrients/components may not necessarily reflect a lack of content validity. For example, *trans*-fat was not considered in the FSANZ and HCST models, possibly because several voluntary initiatives introduced in Australia/New Zealand and Canada during the early 2000s were successful in reducing *trans*-fat levels in the food supply^(^ ^40^ ^,^ ^41^ ^)^. Moreover, the inclusion of nutrients/components is largely dependent on the availability of nutrient composition data^(^ ^1^ ^)^.

With respect to construct/convergent validity, our findings related to the FSANZ and Ofcom models were consistent with another study in that there was overall ‘near perfect’ agreement between the models, other than for fats/oils and dairy products, which were classified differently by the models^(^ ^42^ ^)^. Although the overall discordance observed between the FSANZ and Ofcom model was statistically significant, such a low proportion of discordance (i.e. 5·3 % of foods) is not considered to be of practical significance; rather, this result is meaningful in the context of establishing construct/convergent validity between FSANZ and Ofcom when compared with the overall discordance observed for several of the other models, which ranged from 22·0 to 37·0 %. The ‘slight’ agreement and high proportions of discordance observed for fats/oils and potatoes/sweet potatoes/yams may be explained by the differences in the characteristics of the two models, such as the inclusion of fats in a third food category with a different pre-determined cut-off score and the inclusion of potatoes and tubers as part of FVNL in the FSANZ model but not in the Ofcom model.

Similarly, there was overall ‘near perfect’ agreement between the Nutri-Score and Ofcom model, and the overall discordance observed between the models was low at only 8·3 % of the foods (albeit statistically significant). The ‘fair’ agreement, high proportion of discordance and high proportion of disagreement in cross-classification observed for fruit/fruit juices may be explained by Nutri-Score’s alternative set of criteria, compared with Ofcom, for the attribution of points for the energy, sugar and FVNL content present in specific beverages, such as fruit juices, nectars and smoothies. It should be noted that in order to allow for cross-comparisons in this study, we defined the dichotomous outcomes ‘healthier’ to include foods classified as green-coloured (i.e. dark green (A) or light green (B)) and ‘less healthy’ to include foods not classified as green-coloured (i.e. yellow (C), light orange (D) or dark orange (E)); such categorisation represents a more stringent scenario. Alternatively, if ‘healthier’ foods were defined to also include foods classified as yellow (C), the congruency between the Nutri-Score and Ofcom model determined using the dichotomous outcomes may be affected. Although our findings related to the FSANZ and Nutri-Score models were expected given that these models are derivatives of the Ofcom model, the results nonetheless confirmed construct/convergent validity between these models and Ofcom when used to assess the nutritional quality of pre-packaged foods available in the Canadian marketplace.

The case for construct/convergent validity with the Ofcom model was moderately convincing for the EURO model and less convincing for the PAHO and HCST models. Limited data on the validity of the WHO EURO and PAHO models were identified from the peer-reviewed literature, as the data identified were primarily related to the pilot testing conducted during the models’ development process. The EURO model underwent expert consultation and field-testing using 10 country-specific food composition databases, each of which contained approximately 200 foods that were commonly consumed and/or advertised to children^(^ ^10^ ^,^ ^25^ ^)^. To date, the EURO model has not been tested for construct/convergent validity with healthful diets or predictive validity with health outcomes^(^ ^10^ ^)^. Nevertheless, the EURO model has been adapted for use by other WHO Regional Offices, including the office for the Western Pacific in 2016^(^ ^43^ ^)^, South-East Asia in 2017^(^ ^44^ ^)^ and the Eastern Mediterranean in 2017^(^ ^45^ ^)^. The development of these other WHO models underwent similar testing using country-specific food databases in their respective regions^(^ ^43^ ^–^ ^45^ ^)^. Our findings related to the EURO and Ofcom models were consistent with other studies in that there was overall ‘moderate’ agreement^(^ ^46^ ^)^, and that fats/oils and fruit juices were often classified differently by the models^(^ ^10^ ^)^. For example, there was only ‘slight’ agreement (*κ*=0·08) between the EURO and Ofcom model with a high level of discordance for 62·7 % (*n*682 of 1088) of the fruit/fruit juices, the majority of which were fruit juices specifically (63·0 %, *n*430 of 682). This is possibly because the EURO model considers juices as exclusions, such that they are automatically considered as ‘less healthy’ irrespective of their nutrient content. The healthfulness of juices is viewed differently in various jurisdictions; for example, the UK *Eatwell Guide* recommends that consumption of fruit juices and/or smoothies should be limited to ≤150 ml/d as they are a source of free sugars^(^ ^47^ ^)^, whereas the *2015–2020 Dietary Guidelines for Americans* states that 100 % fruit juices without added sugars and vegetable juices can be part of healthy eating patterns^(^ ^48^ ^)^. As the healthfulness of juices is currently under debate, it remains to be seen whether the consideration of juices in NP models will change.

Although the development of the PAHO model consisted of a comparison with three other NP models (i.e. Ofcom, EURO and a draft of the model for the Eastern Mediterranean region) for the classification of 1992 pre-packaged foods from five European countries, parameters related to agreement or discordance between the models were not assessed, and validity was not discussed^(^ ^26^ ^)^. When PAHO was compared with the Ofcom model in our study, there was only ‘fair’ agreement (*κ*=0·28) and discordance for 33·4 % of the foods overall. For example, agreement was negligible (*κ*=0·01) with a high level of discordance for 44·4 % (*n*80 of 180) of packaged legumes, all of which were classified as ‘less healthy’ by PAHO and ‘healthier’ by Ofcom. For the majority of these legume products (86·3 %, *n*69 of 80), their ‘less healthy’ status was triggered because the Na threshold was exceeded. This discrepancy in classifying legumes is possibly because the Na criterion is calculated per kJ (kcal) of the food according to the PAHO model, whereas it is calculated per 100 g of the food according to the Ofcom model.

On the basis of the cross-classification analyses conducted between the HCST and Ofcom model in our study, adjustments to the nutrient criteria for the food categories with the highest proportions of food classifications that disagreed and/or were grossly misclassified (i.e. eggs, fats/oils, combination dishes, miscellaneous items and sauces/dips/gravies/condiments) may be prudent before the continued use of the HCST model in public health policies, which may in fact coincide well with the revision of CFG that is currently underway^(^ ^49^ ^)^. For example, 75·0 % (*n* 42 of 56) of the classifications for eggs disagreed because they were classified as HCST tier 3 foods (i.e. partially in line with CFG such that fewer choices should come from this tier), but had the lowest quartile of Ofcom scores (i.e. ‘healthiest’ foods). For the majority of these egg products (73·8 %, *n* 31 of 42), their tier 3 status was triggered only because the saturated fat threshold was exceeded, and not because the thresholds for Na or sugars were exceeded. In fact, although the saturated fat content ranged from 2·8 to 3·3 g per CFG serving, these thirty-one egg products were protein-rich sources that provided 10·9–13·2 g of protein per CFG serving. This discrepancy may be explained by the consideration of positive nutrients, such as protein, by the Ofcom model, but not the HCST model.

There are several strengths and limitations associated with this study. First, we recognise that there are other NP models developed by authoritative bodies that could have been examined for content and construct/convergent validity in this study. For example, the Chilean ‘stop sign’ warning label^(^ ^50^ ^)^ was not examined because the Chilean food supply was considered to be different from that in Canada, although data have shown shifts in the Chilean diet characterised by increased consumption of energy-dense foods^(^ ^51^ ^)^; in contrast, as a derivative of the FSANZ model, the Australian Health Star Rating^(^ ^52^ ^)^ was considered to be very similar, and both models were developed and tested for use in the same region. Moreover, a limitation of assessing validity by comparing NP models is the lack of a ‘gold-standard’ measure for defining a ‘healthier’ food^(^ ^17^ ^)^. Although validity testing ideally should involve a ‘gold-standard’ comparator, its necessity in the context of NP has been debated^(^ ^14^ ^,^ ^16^ ^–^ ^18^ ^)^. The method involving the comparison of several models to a validated model, such as Ofcom, is recognised by the WHO^(^ ^1^ ^)^ and others^(^ ^46^ ^,^ ^53^ ^,^ ^54^ ^)^ to be a valid approach. It should be emphasised that we did not consider the Ofcom model to be ‘gold standard’, nor models that differed from Ofcom to be invalid. Rather, our intent was to use the most extensively validated NP model currently available as an objective measure of healthfulness and as one method of identifying components of other models that may warrant further investigation. Nevertheless, a limitation of this method is that the scientific underpinnings of the Ofcom model were established more than a decade ago when the model was developed in 2004 to 2005, and the science of NP has evolved since then. In fact, the Ofcom model is currently being reviewed by Public Health England to ensure that the model reflects the latest dietary guidance^(^ ^55^ ^)^. In addition, validated models other than Ofcom may be suitable as reference models, so cross-comparisons between models can be informative and should be considered in future studies.

Furthermore, a single validation study does not make an NP model valid because different methods of validity testing are associated with various limitations^(^ ^1^ ^,^ ^16^ ^)^. For example, examining the associations between the healthfulness of foods consumed by individuals, as determined using the NP model, and prospective changes in health outcomes is considered the most robust assessment of validity (i.e. predictive); however, this method is complex, costly in time and resources and remains susceptible to recall bias from self-reported dietary recalls^(^ ^1^ ^,^ ^16^ ^)^. This begs the question of whether the demonstration of predictive validity alone would suffice for a model to be considered valid. In fact, there is currently no consensus on a definition with regard to when a model is considered valid. Although Townsend^(^ ^16^ ^)^ and Cooper *et al*.^(^ ^14^ ^)^ echo the sentiment that multiple validation studies using different comparison measures are required to validate one model, the number of studies was not specified. Thus, the scientific standard required to establish that a NP model is sufficiently valid remains unclear at this time.

Typically, other validation studies have used a small sample of several hundred indicator foods across several food categories of interest, data from food composition databases in which the highly aggregated food categories do not allow for variability across similar foods or different brands to be ascertained^(^ ^1^ ^)^ or a single parameter to assess construct/convergent validity (e.g. agreement assessed using the *κ* statistic). To our knowledge, this is the first study to assess the validity of several models by using them to classify over 15 000 foods across numerous food categories, data from a national, branded database that is reflective of the foods available in the marketplace and a combination of parameters to assess construct/convergent validity. Across all foods, the overall proportions of ‘healthier’ and ‘less healthy’ foods were similar in magnitude (all approximately 50 %) between FSANZ and HCST *v.* Ofcom, and a significant positive association was observed between each model and Ofcom. However, the *κ* statistic indicated vast differences in the agreement with Ofcom (‘near perfect’ for FSANZ, but only ‘fair’ for HCST), and the discordance parameter indicated vast differences in the classifications between each model and Ofcom (only 5·3 % for FSANZ, but 37·0 % for HCST). This study highlights the importance of examining a combination of parameters to avoid potential misinterpretation of the results, and the importance of examining classifications across food categories, the level at which differences between models become apparent.

The results of this study may be informative for regulators who wish to adapt and validate existing models for use in country-specific applications. As part of the process in deciding which model to adapt and implement within a jurisdiction, further validation of the model specific to the application, population and legislative framework of that jurisdiction will need to be conducted in order to determine the appropriate level of strictness of the model. This research is conducted as the WHO and many regulatory agencies are working to establish transparent and reproducible methods to underpin their policies aimed at curbing the obesity epidemic and preventing non-communicable diseases. In the span of a year, the Canadian government has proposed to use NP in their policies on front-of-pack labelling and restrictions on the marketing of unhealthy foods to children^(^ ^56^ ^)^. Research specifically on validity testing is timely and globally relevant, as the number of NP models has proliferated, but evidence on the adequacy of these models has lagged behind. As several researchers have suggested that validity testing should be given the highest priority in this field of research^(^ ^10^ ^,^ ^14^ ^,^ ^15^ ^)^, this study contributes to addressing this need.

## Supplementary material

For supplementary material accompanying this paper visit http://dx.doi.org/10.1017/S0007114518001575. click here to view supplementary material
