# Supplementary material for: Comparison of nutrient profiling models for assessing the nutritional quality of foods: a validation study
Source: Br J Nutr. 2018 Jul 17;120(5):567–82. doi: 10.1017/S0007114518001575 (PMC6137431; doi:10.1017/S0007114518001575)
Supplement: Supplementary file 1 [file S0007114518001575sup001.pdf]

Supplementary Table 1. Definitions of different types of validity used by different researchers

Type of validity	Definition(s)	Reference(s)
Criterion	Extent to which the measurement correlates with an external criterion of the phenomenon under study; ideally, a gold standard[a,b], but it need not[c].	Porta, 2014[a]<br>Arambepola *et al*., 2008[b]<br>Scarborough *et al*., 2007[c]
	Extent to which the method is accurately based on an externally derived gold standard; examines whether method correlates in a predicted manner with variables with which, theoretically, it should correlate.	Townsend, 2010[d]
	Accuracy of the NP model scores based on an externally derived objective measure of healthfulness.	Cooper *et al*., 2016[e] (citing Townsend, 2010[d])
Predictive (type of criterion)	Extent to which the measurement is able to predict an external criterion of the phenomenon under study.[f]	Porta, 2014[a]<br>Rayner, 2011 (unpublished manual)[g] (citing Porta, 2014[a])
	Independent criterion measure is obtained after the test score.	Cronbach and Meehl, 1955[h]
	Extent to which the system reflects a population's or an individual's diet change over time; extent to which the system reflects a change in nutritional and health status.	Townsend, 2010[d]
	Ability of the tool to predict future outcomes, in which case the tests are performed at different times and then the correlation between the result of the tool and the secondary outcome is determined.	Cooper *et al*., 2016[e]
Concurrent (type of criterion)	Measurement and criterion refer to the same point in time.[i]	Porta, 2014[a]<br>Cronbach and Meehl, 1955[h]
	Requires that the tool be tested against some other method acknowledged as a gold standard for assessing the same variable, and the two tests are conducted at the same time.	Cooper *et al*., 2016[e]
Convergent	Extent to which the system is accurately based on self-report (internally derived) data with hypothesised relation; examines whether method correlates in a predicted manner with variables with which, theoretically, it should correlate.	Townsend, 2010[d]
	Extent to which the measurement correlates with an external criterion of the phenomenon under study at the same point in time.	Rayner, 2011 (unpublished manual)[g] (citing Porta, 2014[a])
	Comparison with other measures, not necessarily better measures, of the same variable or a closely related variable.	Arambepola *et al*., 2008[b]
Discriminant	Extent to which the system discriminates between groups expected to be different.	Townsend, 2010[d]
	Comparison with other measures, not necessarily better measures, of variables that are not closely related.	Arambepola *et al*., 2008[b]
Construct	Extent to which the measurement corresponds to theoretical concepts (constructs) concerning the phenomenon under study.[j]	Porta, 2014[a]<br>Arambepola *et al*., 2008[b] (citing Cronbach and Meehl, 1955[h])<br>Rayner, 2011 (unpublished manual)[g] (citing Porta, 2014[a])

Supplementary Table 1. Definitions of different types of validity used by different researchers

Type of validity	Definition(s)	Reference(s)
	Comparison with other measures of the same variable, or closely related ones.	Cooper *et al.*, 2017[k] (citing Arambepola *et al.*, 2008[b] and WHO, 2010[l])
	Correlation between how the NP model ranks the healthfulness of foods in comparison to other measures that determine the healthfulness of food.	Cooper *et al.*, 2016[e] (citing Arambepola *et al.*, 2008[b] and Townsend, 2010[d])
Content	Extent to which the measurement incorporates the domain of the phenomenon under study.[m]	Porta, 2014[a] Rayner, 2011 (unpublished manual)[g] (citing Porta, 2014[a])
	Extent to which the system covers the full range of meaning for the concept being measured; assessment of the consistency between the science underlying the algorithms and the science published in the peer-reviewed literature.	Townsend, 2010[d]
	Consideration and analysis of the components that make up the NP model with reference to current scientific literature.	Cooper *et al.*, 2016[e] (citing Townsend, 2010[d])
Face	Extent to which a measurement or a measurement instrument appears reasonable on superficial inspection.	Porta, 2014[a]
	Extent to which the system is a useful tool to the consumer making food-purchase decisions in the marketplace; determined by end users of the system (*i.e.*, the consumer).	Townsend, 2010[d] Cooper *et al.*, 2016[e]

NP, nutrient profiling.

[a] Porta M (2014) A Dictionary of Epidemiology, 6th ed., [S Greenland, M Hernán, I dos Santos Silva and JM Last, editors]. New York: Oxford University Press.

[b] Arambepola C, Scarborough P, Rayner M (2008) Validating a nutrient profile model. Public Health Nutr 11, 371-378.

[c] Scarborough P, Boxer A, Rayner M et al. (2007) Testing nutrient profile models using data from a survey of nutrition professionals. Public Health Nutr 10, 337-345.

[d] Townsend MS (2010) Where is the science? What will it take to show that nutrient profiling systems work? Am J Clin Nutr 91, 1109S-1115S.

[e] Cooper SL, Pelly FE, Lowe JB (2016) Construct and criterion-related validation of nutrient profiling models: a systematic review of the literature. Appetite 100, 26-40.

[f] An example is an academic aptitude test that is validated against subsequent academic performance (Porta, 2014).

[g] Rayner (unpublished) Guiding Principles and Framework Manual for the Development or Adaptation of Nutrient Profile Models (First Edition (2011)). Geneva: WHO.

[h] Cronbach LJ, Meehl PE (1955) Construct validity in psychological tests. Psychol Bull 52, 281-302.

[i] An example is a visual inspection of a wound for evidence of infection validated against bacteriological examination of a specimen taken at the same time (Porta, 2014).

[j] For example, if on theoretical grounds the phenomenon should change with age, a measurement with construct validity would reflect such a change (Porta, 2014).

[k] Cooper SL, Pelly FE, Lowe JB (2017) Assessment of the construct validity of the Australian Health Star Rating: a nutrient profiling diagnostic accuracy study. Eur J Clin Nutr, 1-7.

[l] World Health Organization (2010) Nutrient Profiling. Report of a WHO/IASO Technical Meeting, London, United Kingdom 4–6 October 2010. Geneva: WHO. http://www.who.int/nutrition/publications/profiling/WHO_IASO_report2010.pdf.

[m] For example, a measurement of functional health status should embrace activities of daily living (*e.g.*, occupational, family, and social functioning) (Porta, 2014).

Supplementary Table 2. Method based on the ingredient list used to estimate the FVNL points of foods in the Ofcom model[a]

Ofcom criteria		
Point(s)	FVNL content of food (%)	In-house criteria based on the ingredient list used to estimate FVNL content[b]
Non-concentrated FVNL ingredients		
0	≤40	FVNL is not one of the first two ingredients.
1	>40	FVNL is the second ingredient.
2	>60	FVNL is the first ingredient, but non-FVNL ingredients appear to contribute substantially to the product's weight[c].
5	>80	FVNL is the first ingredient, and only FVNL ingredients contribute substantially to the product's weight.
Concentrated FVNL ingredients[d]		
0	≤40	FVNL is not one of the first three ingredients.
1	>40	FVNL is the second ingredient, but the amounts of the first and second ingredients are not similar (see corresponding criteria for 2 points[e]).
		FVNL is the third ingredient and is estimated to represent ≥25% of the product's weight[f].
2	>60	FVNL is the first ingredient, but non-FVNL ingredients appear to contribute substantially to the product's weight[c].
		FVNL is the second ingredient, and the amounts of the first and second ingredients are similar[e].
5	>80	FVNL is the first ingredient, and only FVNL ingredients contribute substantially to the product's weight.

FVNL, fruits, vegetables, nuts, and legumes.

[a] In the absence of quantitative declarations in the ingredient list, which are not required for food labelling in Canada, a method was developed by the research group at the University of Toronto in order to estimate the FVNL content and corresponding FVNL points of the foods for the Ofcom model. Given that the ingredients are listed in descending order by weight, the presence and positions of the FVNL ingredients within the ingredient list were used to estimate the FVNL content.

[b] If sub-ingredients in brackets were presented for an ingredient, the ingredient was considered a FVNL if one of the first two sub-ingredients was a FVNL. If a food consisted of more than one component, and therefore more than one ingredient list (e.g. tuna kit with crackers), FVNL points were calculated for the individual components, and the average of the FVNL points was used to represent the food as a whole. If the ingredient list of a food was missing (<2% of foods), FVNL points were not assigned unless it was evident from the product name that FVNL contributed substantially to the product's weight.

[c] Non-FVNL ingredients that were considered to contribute minimally to the product's weight included: salt, preservatives, colour, vitamins, minerals, oils, flavour extracts, antioxidants, and food additives. Other non-FVNL ingredients were considered to contribute minimally if they appeared after salt or preservatives in the ingredient list. Non-FVNL ingredients that were considered to contribute substantially to the product's weight were other than those previously listed (e.g. sugar and water were considered to contribute substantially to the product's weight).

[d] Concentrated FVNL ingredients are those in concentrated form (i.e. dried, evaporated, as pastes). According to the Ofcom model, concentrated FVNL contribute less than non-concentrated FVNL to the weight of the food; thus, the weight of concentrated FVNL should be multiplied by two when calculating the FVNL content of a food. Given that this criterion

cannot be applied directly in the absence of quantitative declarations, the criteria were adjusted for concentrated FVNL ingredients.

[e] A food may score two points if the total number of ingredients present in the ingredient list is low (e.g. ≤3), and if it is estimated that the amount of the first ingredient is only slightly higher than the amount of FVNL in the food (e.g. estimated proportion of 55 *versus* 45%, respectively).

[f] A food may score one point because at least 25% of the weight represented by a concentrated FVNL equals to at least 40% FVNL when multiplying the amount by two according to the Ofcom model.

Supplementary Table 3. Proportion (%) of "healthier" and "less healthy" foods classified by models compared to the Ofcom model for all foods (n=15,227) and by food category

Schedule M category and description	Foods (n)	Ofcom	FSANZ "Healthier"	FSANZ "Less healthy"	Nutri-Score "Healthier"	Nutri-Score "Less healthy"	HCST "Healthier"	HCST "Less healthy"	EURO "Healthier"	EURO "Less healthy"	PAHO "Healthier"	PAHO "Less healthy"
All	15,156 to 15,183[a]	"Healthier"	44.1	0.3	36.2	8.2	29.0	15.4	26.1	18.3	13.4	31.0
		"Less healthy"	5.0	50.6	0.0	55.6	21.6	34.0	3.7	52.0	2.4	53.2
1. Bakery products	2082 to 2083	"Healthier"	24.2	0.0	22.6	1.5	23.9	0.2	13.9	10.3	3.1	21.0
		"Less healthy"	3.5	72.4	0.1	75.8	39.5	36.3	0.9	75.0	0.2	75.7
2. Beverages	481 to 482	"Healthier"	49.2	1.0	42.3	7.9	47.9	2.3	11.4	38.9	9.5	40.7
		"Less healthy"	5.6	44.2	0.0	49.8	9.1	40.7	0.0	49.7	0.0	49.8
3. Cereals, other grains	978 to 981	"Healthier"	75.5	0.0	74.6	0.9	75.0	0.5	69.5	6.0	67.2	8.4
		"Less healthy"	6.1	18.4	0.0	24.5	22.4	2.2	1.8	22.6	1.0	23.5
4. Dairy products, substitutes	1,237	"Healthier"	37.1	0.1	22.1	15.1	27.8	9.4	16.3	20.9	6.1	31.1
		"Less healthy"	17.5	45.3	0.0	62.8	8.3	54.5	5.2	57.6	2.4	60.4
5. Desserts	820 to 827	"Healthier"	32.3	0.7	20.0	13.1	24.0	9.3	0.0	33.0	0.1	32.9
		"Less healthy"	0.6	66.4	0.1	66.9	6.0	60.7	0.0	67.0	0.0	67.0
6. Dessert toppings, fillings	115	"Healthier"	11.3	1.7	6.1	7.0	8.7	4.4	0.0	13.0	0.0	13.0
		"Less healthy"	0.0	87.0	0.0	87.0	37.4	49.6	0.0	87.0	0.9	86.1
7. Eggs	56	"Healthier"	94.6	0.0	94.6	0.0	19.6	75.0	94.6	0.0	82.1	12.5
		"Less healthy"	0.0	5.4	0.0	5.4	0.0	5.4	5.4	0.0	0.0	5.4
8. Fats, oils	535	"Healthier"	1.9	0.0	1.7	0.2	1.9	0.0	0.4	1.5	0.4	1.5
		"Less healthy"	33.8	64.3	0.0	98.1	66.7	31.4	28.2	69.9	27.3	70.8
9. Marine products	440	"Healthier"	77.3	0.0	70.5	6.8	48.2	29.1	75.5	1.8	14.6	62.7
		"Less healthy"	0.0	22.7	0.0	22.7	2.7	20.0	9.6	13.2	0.2	22.5
10. Fruit, fruit juices	1,088	"Healthier"	69.8	2.7	33.9	38.5	59.6	12.9	9.7	62.7	38.8	33.6
		"Less healthy"	1.3	26.3	0.0	27.6	16.4	11.2	0.0	27.6	3.1	24.5

Supplementary Table 3. Proportion (%) of "healthier" and "less healthy" foods classified by models compared to the Ofcom model for all foods (n=15,227) and by food category

Schedule M category and description	Foods (n)	Ofcom	FSANZ		Nutri-Score		HCST		EURO		PAHO	
			"Healthier"	"Less healthy"	"Healthier"	"Less healthy"	"Healthier"	"Less healthy"	"Healthier"	"Less healthy"	"Healthier"	"Less healthy"
11. Legumes	180	"Healthier"	99.4	0.0	99.4	0.0	81.7	17.8	85.0	14.4	55.0	44.4
		"Less healthy"	0.0	0.6	0.0	0.6	0.0	0.6	0.0	0.6	0.0	0.6
12. Meat, poultry, their products, substitutes	895	"Healthier"	27.3	0.1	22.8	4.6	13.6	13.7	25.9	1.5	0.9	26.5
		"Less healthy"	0.6	72.1	0.0	72.6	0.3	72.3	16.3	56.3	0.5	72.2
13. Miscellaneous	444 to 445	"Healthier"	20.9	0.0	16.0	4.9	21.0	0.0	7.6	13.3	10.1	10.8
		"Less healthy"	1.6	77.5	0.0	79.1	63.3	15.8	0.2	78.9	3.4	75.7
14. Combination dishes	1,347 to 1,348	"Healthier"	71.6	0.0	61.4	10.2	8.0	63.6	50.5	21.1	2.0	69.6
		"Less healthy"	1.2	27.2	0.0	28.4	2.1	26.4	1.6	26.8	0.0	28.4
15. Nuts, seeds	220	"Healthier"	65.5	0.0	62.3	3.2	47.7	17.7	47.7	17.7	62.7	2.7
		"Less healthy"	10.5	24.1	0.0	34.6	4.1	30.5	5.9	28.6	12.3	22.3
16. Potatoes, sweet potatoes, yams	140	"Healthier"	67.9	0.0	55.0	12.9	49.3	18.6	32.1	35.7	12.9	55.0
		"Less healthy"	27.9	4.3	0.0	32.1	4.3	27.9	7.1	25.0	0.0	32.1
17. Packaged salads	70	"Healthier"	75.7	0.0	68.6	7.1	27.1	48.6	38.6	37.1	0.0	75.7
		"Less healthy"	5.7	18.6	0.0	24.3	0.0	24.3	4.3	20.0	0.0	24.3
18. Sauces, dips, gravies, condiments	1,219 to 1,224	"Healthier"	31.1	0.0	27.4	3.8	16.2	15.0	9.6	21.5	2.6	28.5
		"Less healthy"	0.3	68.6	0.0	68.9	41.4	27.3	1.9	67.0	1.5	67.4
19. Snacks	794	"Healthier"	17.6	0.1	14.5	3.3	4.8	13.0	5.5	12.2	2.6	15.1
		"Less healthy"	10.6	71.7	0.0	82.2	16.4	65.9	1.5	80.7	1.4	80.9
20. Soups	454 to 456	"Healthier"	89.3	0.0	73.5	15.8	7.9	81.5	86.8	2.4	1.3	87.9
		"Less healthy"	0.2	10.5	0.0	10.8	0.0	10.6	6.4	4.4	0.0	10.8
21. Sugars, sweets	739	"Healthier"	3.5	0.0	3.0	0.5	3.5	0.0	0.5	3.0	0.0	3.5
		"Less healthy"	0.1	96.4	0.0	96.5	37.4	59.1	0.0	96.5	8.8	87.7

Supplementary Table 3. Proportion (%) of "healthier" and "less healthy" foods classified by models compared to the Ofcom model for all foods (n=15,227) and by food category

Schedule M category and description	Foods (n)	Ofcom	FSANZ		Nutri-Score		HCST		EURO		PAHO	
			"Healthier"	"Less healthy"	"Healthier"	"Less healthy"	"Healthier"	"Less healthy"	"Healthier"	"Less healthy"	"Healthier"	"Less healthy"
22. Vegetables	827 to 828	"Healthier"	70.7	0.1	66.1	4.7	65.7	5.1	48.8	22.0	31.9	38.9
		"Less healthy"	0.1	29.1	0.0	29.2	24.8	4.5	0.0	29.2	0.0	29.2

FSANZ, Food Standards Australia New Zealand; HCST, Health Canada Surveillance Tool; EURO, World Health Organization Regional Office for Europe; PAHO, World Health Organization Regional Office for the Americas/Pan American Health Organization.

[a] Across the models, data were missing for 0.29 to 0.41% (n=44 to 62) of foods.
